# A Bill for a Medical Examining Board

**Published:** 1892-12

**Authors:** 


### *EDITORIAL.*

---

The office of THE JOURNAL is in room 330 Equitable Building.
Address communications relating to the Editorial Department to DR. L. B. GRANDY, Atlanta, Ga., Box 431.
Business communications should be addressed to DR. M. B. HUTCHINS, Atlanta, Ga.
All remittances should be made payable to "ATLANTA MEDICAL AND SURGICAL JOURNAL."
Articles for publication should reach this office not later than the 15th instant.
The editors are not responsible for views expressed by contributors.

---

### A BILL FOR A MEDICAL EXAMINING BOARD.

---

We are pleased to announce that a bill for the establishment of a Medical Examining Board has been prepared and submitted to the General Assembly. The committee of able gentlemen who constructed it, prominent representatives of the Atlanta medical profession, have performed their work with zeal and dispatch, carefully consulted the medical practice acts of other States, and framed a measure which deserves to become a law in Georgia. It is free from objectionable features, conservative and fair. The bill is now before the Senate, where its friends are encouraged to hope for a favorable consideration. Of course it will recieve opposition from certain quarters, but those who wish to see quackery expelled, as far as possible, from the State, the profession elevated, the cause of medical education advanced, and Georgia no longer the secure resting-place for those who have been rejected in other communities as either unworthy or incompetent, will give this matter their cordial support.

But here is the bill:

A Bill to be entitled an Act to establish a Board of Medical Examiners for the State of Georgia, and to protect the people from illegal and unqualified practitioners of medicine.

SECTION 1. Be it enacted by the General Assembly of the State of Georgia, That within ninety days after the passage of this Act it shall be the duty of the Governor to appoint for this State a Board of Medical Examiners consisting of one member from each congressional district in this State and five from the State at large, of whom one shall be a homeopathic practitioner and two shall be eclectic practitioners. The term of office of said members shall be for three years; *provided*, that five shall be first appointed for one year, five for two years and six for three years, and subsequently each appointment shall be for the full term of the three years. The members of said board shall be men learned in medicine and surgery and of good moral and professional character, and none of them shall be members of the faculty of any medical college. Any vacancy that may occur in said board in consequence of death, resignation or removal from the State or district shall be filled for the unexpired term by the Governor.

SEC. 2. Be it further enacted, That immediately and before entering upon the duties of said office, the members of said board shall take the following oath: I do swear that I will support the Constitution of this State and the United States and faithfully perform the duties of a member of the Board of Medical Examiners for the State of Georgia to the best of my ability, so help me God; and shall file the same in the office of the Secretary of State, who, upon receiving the said oath of office, shall issue to each examiner a certificate of appointment.

SEC. 3. Be it further enacted, That immediately after the appointment and qualification of said members they shall meet and organize as a Board of Medical Examiners. The officers shall be a President, a Vice-President and a Secretary (who shall act as Treasurer), said officers to be members of and elected by the board. Regular meetings of the board shall be held at such times and places as the board may prescribe and special meetings may be held upon the call of the President and three members, but there shall not be less than one regular meeting each year. Nine (9) members of the board shall constitute a quorum. The board may prescribe rules, regulations and by-laws for its own proceedings and government and for the examination by its individual members of candidates for the practice of medicine and surgery.

SEC. 4. Be it further enacted, That it shall be the duty of said board at any of its meetings to examine all persons making application to it who are graduates of any incorporated medical college, school or university that requires three (3) full courses of study of six months each, who shall desire to commence the practice of medicine or surgery in this State and who shall not, by the provisions of this Act, be exempt from such examinations. But any person now matriculated as a student of medicine at any medical college, after graduation, and any person from another State who shall have graduated prior to April 1, 1893, at a lawfully chartered medical college requiring only two full courses of study shall be examined and licensed as herein provided. When an applicant shall have passed an examination satisfactory as to proficiency before the board in session, the President thereof shall grant to such applicant a certificate to that effect. A fee of fifteen ($15) dollars shall be paid to such board through such officer or member as it may designate by each applicant before such examination is had.

In case an applicant shall fail to pass a satisfactory examination before the board he shall not be permitted to stand any further examination within the next three (3) months thereafter. Nor shall he have again to pay the fee prescribed as aforesaid ; *provided, however*, that no applicant shall be rejected upon his examination on account of his adherence to any particular school of medicine or system of practice, nor on account of his views as to the method of treatment and cure of diseases; *and provided further*, that when, in the opinion of the President of the board, any applicant has been prevented by good cause from appearing before the board the President of the board shall appoint a committee of three members who shall examine such applicant and may, if they see fit, grant him a certificate which shall have the same force and effect as though granted by the full board, until the applicant shall have an opportunity to appear before the said board, when, if the applicant fails to appear for examination, the President of the board shall have authority to revoke such certificate, or, in any case, the President shall have authority at his discretion to grant a special permit to any applicant to practice medicine until he shall have an opportunity to appear before the board in session for examination, which said special permit shall be revokable at the discretion of the President.

SEC. 5. Be it further enacted, The fund realized from the fees aforesaid shall be applied by the board to the payment of its expenses and to making a reasonable compensation to the President, Secretary and members thereof.

SEC. 6. Be it further enacted, That before any person who obtains a certificate from the board may lawfully practice medicine or surgery in this State he shall cause the said certificate to be recorded in the office of the clerk of the Superior Court in the county in which he resides. But, if he does not reside in the State of Georgia he shall cause said certificate to be recorded in the county within this State in which he offers to practice. The certificate shall be recorded by the clerk in a book to be kept for that purpose. It shall be indexed in the name of the person to whom the certificate is granted. The clerk's fee for recording the certificate shall be the same as for recording a deed.

Sec. 7. Be it further enacted, That this Act shall take effect from the first day of April, 1893, and that it shall be unlawful thereafter for any person to commence the practice of medicine or surgery in this State without first complying with the provisions of this Act. But nothing in this Act shall apply to persons now residents of this State lawfully engaged in the practice of medicine or surgery in the State of Georgia, to any commissioned medical officer or contract surgeon of the United States army or navy or of the Marine hospital service in the performance of their duties as such nor to any physician or surgeon residing in any State or territory of the United States or in the District of Columbia who may be called in consultation in a special case with a physician or surgeon residing in this State, or to any midwife or dentist in the practice of their vocation, nor shall this Act be construed as affecting or changing in any way laws in reference to the license tax to be paid by physicians, surgeons and dentists.

Sec. 8. Be it further enacted, That any person shall be regarded as practicing medicine or surgery within the meaning of this Act who shall profess publicly to be a physician or surgeon and shall offer to practice as such, or who shall prescribe for the sick or those needing medical or surgical aid, and shall charge or receive therefor money or other compensation or consideration, directly or indirectly.

Sec. 9. Be it further enacted, That any person who shall practice medicine or surgery in this State in violation of the provisions of this Act shall, upon conviction, be fined not less than fifty dollars nor more than five hundred dollars for each offence, and it shall not be lawful for him to recover by action, suit, motion or warrant any compensation for services which may be claimed to have been rendered by him as such physician or surgeon.

Sec. 10. Be it further enacted, That all laws and parts of laws in conflict with this Act be, and the same are, hereby repealed.

As we go to press we learn that the above bill, after a very feeble opposition, passed the Senate by a vote of thirty-five to nine. It now goes into the House.

